# Quantification and stimulation of human glymphatic dynamics: New features of Alzheimer’s disease and effects of brain photobiomodulation

**DOI:** 10.21203/rs.3.rs-6115809/v1

**Published:** 2025-03-05

**Authors:** Fiza Saeed, Kathy L. Siepker, Soeun Jang, Sadra Shahdadian, Hanli Liu

**Affiliations:** University of Texas at Arlington; University of Texas at Arlington; University of Texas at Arlington; University of Texas at Arlington; University of Texas at Arlington

**Keywords:** broadband near-infrared spectroscopy, human glymphatic dynamics, features of Alzheimer’s disease, transcranial photobiomodulation, cerebrospinal fluid

## Abstract

A non-invasive device to measure the dynamics of cerebrospinal fluid (CSF) is highly desirable because CSF facilitates the cleaning of neurotoxic wastes in the brain. A better understanding of CSF dynamics helps promote healthy aging in older adults and to treat patients with neurological diseases. This study employed a multi-color optical method to quantify prefrontal CSF dynamics in two groups: (1) older adults with (n = 16) and without (n = 27) Alzheimer’s disease and (2) young adults (n = 26) before and after prefrontal light stimulation. The results revealed that the coupling strengths between cerebral blood volume (CBV) and CSF were age-dependent and significantly higher in AD patients than in healthy controls. Prefrontal light stimulation significantly enhanced CBV-CSF coupling, suggesting improved CSF drainage. This study underscores the multi-color optical strategy as a unique tool for monitoring the interaction between CBV and CSF, as well as metabolic functions in the human brain, while demonstrating the therapeutic potential of brain light stimulation in treating neurodegenerative diseases involving CSF drainage dysfunction.

## TEASER

This study shows an all-optics strategy, one of which characterizes glymphatic dynamics in patients with Alzheimer’s disease and the other promotes glymphatic circulation.

## INTRODUCTION

**Figure 1:**
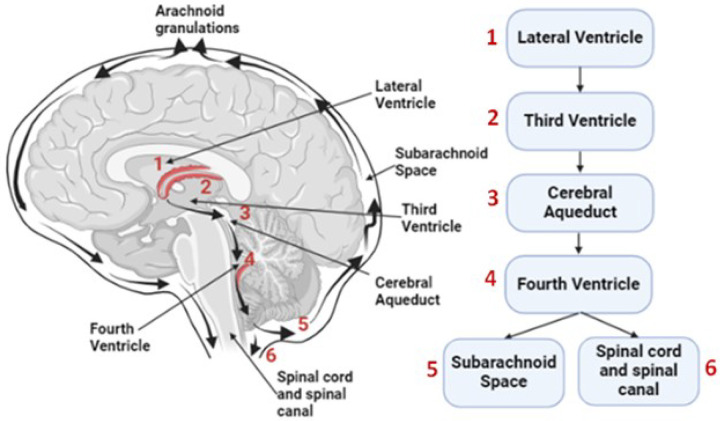
Illustration of CSF flow in the human brain. CSF is generated or initiated from (1) the lateral ventricles, travels towards (2) the third ventricles, flows through (3) cerebral aqueducts, arrives at (4) the fourth ventricles, and then drains into (5) the subarachnoid space and (6) the spinal cord/canal. The arrows indicate the direction of CSF flow originating from the lateral ventricles.

Water serves as an essential support system for human life, making up approximately 73% of the brain and heart volume and approximately 64% of the skin’s composition (1). Among the fluids in the body, cerebrospinal fluid (CSF), which consists of approximately 99% water, is produced and replenished several times daily. CSF plays a vital role in sustaining brain health (2) because its circulation acts as a cleansing mechanism for the interstitial fluid of the brain and removes neurotoxic waste products including degraded proteins and metabolites. This process is part of the glymphatic drainage system, which is a network of spaces in the brain that removes waste and helps distribute other compounds. In particular, the glymphatic drainage system eliminates the accumulation of toxins (3) such as amyloid-β (Aβ), a hallmark of Alzheimer’s disease (AD) and a precursor to neuronal dysfunction. This waste-clearance process is significantly enhanced during sleep due to increased CSF flow (4, 5). The macroscopic pathway of CSF in the brain is shown in Fig. 1. Given the key role played by the glymphatic system in human brain health, it is highly desirable to noninvasively monitor and quantify glymphatic drainage dynamics in patients with neurological diseases and in healthy older adults (OA) wishing for healthy longevity.

A novel analysis, introduced recently by Myllylä et al., employed conventional near-infrared spectroscopy (NIRS) with 2–3 wavelengths to monitor fluctuations of cerebral free water content or CSF, enabling the quantification of glymphatic system dynamics in humans (6). Building on this methodology, a study employing 4-wavelength NIRS identified an inverse linear correlation between normalized alterations in cerebral total hemoglobin concentration (HbT) and free water content in CSF (H_2_O_free_) in 51 healthy participants (7). It is acknowledged that neurological disorders such as dementia are closely associated with glymphatic drainage disruption. Thus, quantifying alterations in coupling between HbT and H_2_O_free_, which represents cerebrovascular-CSF coupling, offers a valuable means to reveal the functional mechanisms driving glymphatic drainage circulation. This new analysis method provides fresh metrics to assess brain health in humans with or without neurological disorders, with or without intervention. For instance, it can help to identify clinically meaningful glymphatic features in patients with AD, which may serve as potential biomarkers for the early diagnosis of AD.

Furthermore, transcranial photobiomodulation (tPBM) using NIR light (800–1070 nm) has been reported as a promising noninvasive intervention or therapeutic strategy to augment cognition in healthy humans or to treat patients with neurological or cognitive impairments (8–10). The established benefits of tPBM include improved metabolism and microcirculation along with reduced oxidative stress and inflammation (9–12). Beyond the positive and significant impacts of tPBM on cerebral hemodynamic and metabolic activities (13–16), recent animal studies have revealed that tPBM using a 1267-nm laser enabled activation of meningeal lymphatic vessels (MLVs) for glymphatic clearance of beta-amyloid from the mouse brain (17, 18), and that tPBM with a 1065-nm LED enhanced the animal brain’s drainage system during sleep that improved learning and memory in male mice (19). All these animal studies supported tPBM as a promising strategy for nonpharmacologic therapy of AD. Its rationale is that tPBM stimulates MLVs, increases oscillations of MLVs, and accelerates the flow of CSF, thus improving the excretion of harmful substances and metabolites, including Aβ, from the brain (5, 20). However, all tPBM-augmented glymphatic clearance were based on animal studies, and no cerebral hydrodynamic coupling in response to tPBM has been investigated in humans.

This study aimed to determine metrics of HbT-CSF coupling in the prefrontal cortex (PFC) using two-channel broadband NIRS (2bbNIRS) (15, 21) for two specific biomedical applications. One application was to explore the significant differences in the coupling metrics of HbT-CSF between healthy older adults (n = 27) and patients with AD (n = 15) for identifiable features of the AD brain. The other application was to examine whether the brains of young adults (n = 26) were stimulated by tPBM to increase the strength of HbT-CSF coupling, as observed in animal studies (17–19). By the end of the study, the results showed that HbT-CSF coupling of the AD brain was significantly stronger than that of its counterparts to compensate for several pathological dysfunctions in AD. For the second application, the study revealed that tPBM with 800-nm and/or 850-nm lasers on the forehead enabled a significant increase in HbT-CSF coupling, implying augmentation of glymphatic drainage function. These results also highlight bbNIRS as a noninvasive monitoring tool to assess multi-neurophysiological parameters for brain health and support the therapeutic potential of tPBM for treating neurodegenerative diseases that require repair of the glymphatic dynamics.

## RESULTS

### Correlations of HbT and CSF in PFC of older adults with and without AD

**Figure 2:**
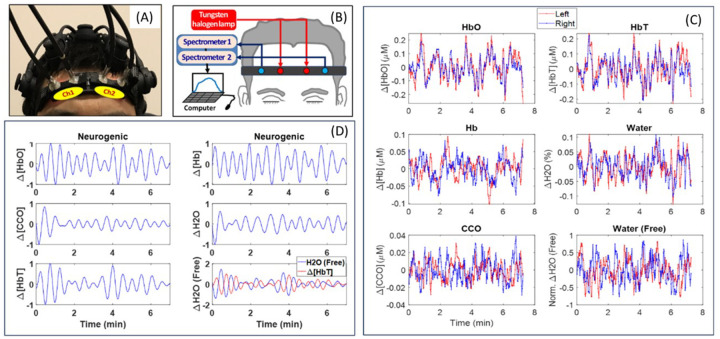
Illustration of the bbNIRS setup and sample results. (A) A photo of two optical probes of a 2bbNIRS device placed on the forehead of a participant. Multiple legs of an electroencephalogram (EEG) on the head were also attached on the head for concurrent EEG readings, which are not the scope of this paper and thus not presented hereafter. (B) A schematic display of the 2bbNIRS setup. (C) An example of a set of time series of D[HbO], D[Hb], D[HbT], D[oxiCCO], D[H_2_O], and normalized D[H_2_O_free_] from a participant. Two temporal files in each sub-panel show the respective readings from the left (red) and right (blue) PFC of the participant. (D) An example of normalized time series of D[HbO], D[Hb], D[HbT], D[oxiCCO], D[H_2_O], and D[H_2_O_free_] (blue) in the neurogenic band. Note that D[H_2_O_free_] time series is plotted together with normalized D[HbT] (red), showing anti-correlated patterns between them.

To determine prefrontal HbT-CSF correlations of human participants, we employed a 2bbNIRS system with two lateral channels placed on the forehead (Figs. 2A and 2B) of healthy older adults (OA; n = 27) and patients with AD (n = 16). For this group of participants, 7-min resting-state measurements across 740–1100 nm were performed concurrently on both lateral sides. Based on the modified Beer-Lambert law, temporal alterations in concentrations of oxy-hemoglobin (Δ[HbO]), deoxy-hemoglobin(Δ[Hb]), total hemoglobin (Δ[HbT] = Δ[HbO] + Δ[Hb]), oxidized cytochrome c oxidase (Δ[oxiCCO]), water content in PFC (Δ[H_2_O]), and free water content in CSF (Δ[H_2_O_free_]) were obtained from each channel (Fig. 2C). Furthermore, all six time series shown in Fig. 2C were normalized between − 1 and 1 after being decomposed into three frequency bands of infraslow oscillation,(22, 23) namely, endogenic (E; 0.005–0.02 Hz), neurogenic (N; 0.02–0.04 Hz), and myogenic (M; 0.04–0.1 Hz) frequencies. As examples, Fig. 2D shows a set of time series in the neurogenic band for each of the six neurophysiological quantities. The sub-panel on the right bottom of Fig. 2D shows temporal profiles of Δ[HbT] (red) and Δ[H_2_O_free_] (blue), exhibiting inverse oscillation pattens between them. Time series in all three E/N/M bands can be found in Figure S1 of Supplementary Materials.

**Figure 3:**
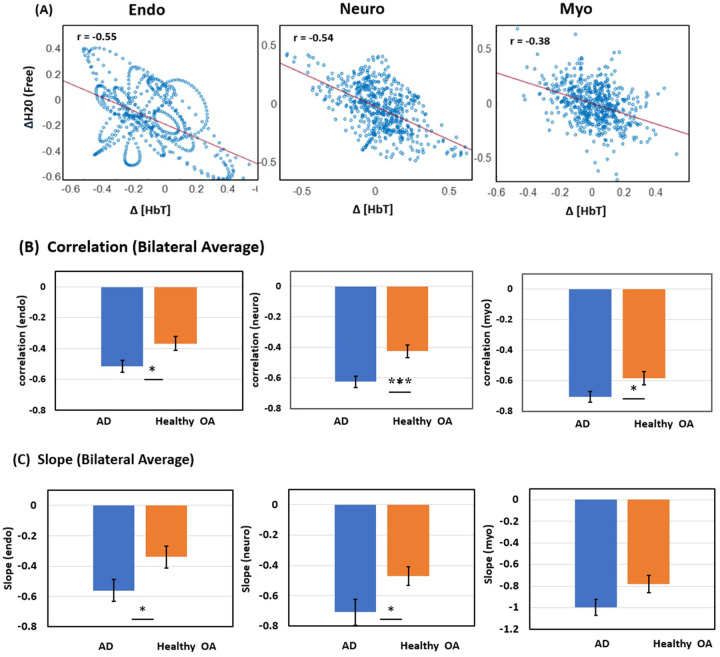
Comparisons between AD patients and healthy older adults (OA) (A) An example of relationships between normalized Δ[HbT] and Δ[H_2_O_free_] with linear correlation fitting in each of the three E/N/M bands from an AD patient. (B) Correlation coefficients and (C) correlation slopes of HbT-CSF coupling in the PFC of AD patients (n=16) and healthy older adults (n=27) in three frequency bands: the endogenic (E; 0.005–0.02 Hz), neurogenic (N; 0.02–0.04 Hz), and myogenic (M; 0.04–0.1 Hz) bands. Error bars were obtained from the SEM of each respective group. *: p < 0.05; **: p < 0.01; ***: p < 0.001 by two-sample Student’s t-test.

Given the inverse oscillation patterns between normalized Δ[HbT] and Δ[H_2_O_free_], linear correlation fitting was performed to assess HbT-CSF coupling in each of the three E/N/M bands (Fig. 3A) for each lateral side of the bbNIRS measurements. This analysis yielded two correlation metrics for each oscillation band: (1) correlation coefficient and (2) correlation slope. To evaluate HbT-CSF coupling metrics at the group level, the same fitting process was applied to both PFC sides for the two sub-groups, healthy OA and patients with AD. By combining the metrics across the two lateral channels within each sub-group, we obtained group-level correlation coefficients (Fig. 3B) and slopes (Fig. 3C) in each of the E/N/M bands of the PFC.

A two-sample t-test was conducted between the two sub-groups in each frequency band (Table 1). The statistical analysis results revealed that the correlation coefficients R (Table 1A) of HbT-CSF coupling in the PFC of AD patients were significantly more negative than those of healthy OA in all E/N/M oscillation bands with medium to large effect sizes, as indicated by Cohen’s *d* (−0.65 to −1.06; Table 1A). Similarly, the correlation slopes of the AD patients were significantly more negative than those of healthy OA in the E/N bands and marginally significant (*p* = 0.052) in the M band, with medium effect sizes across the three bands (−0.6 to −0.72; Table 1B).

### Correlations between HbT and CSF in PFC pre- and post tPBM

**Figure 4:**
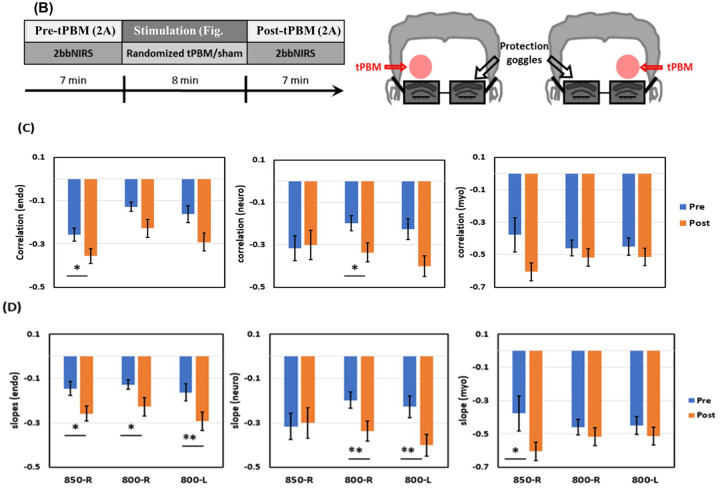
tPBM protocol with setup and tPBM-induced effects (A) Experimental protocol for tPBM with a 7-min pre- and post-stimulation period, during which bilateral 2bbNIRS measurements were performed using the setup shown in Figures 2A and 2B. (B) During the 8-min tPBM, the 2bbNIRS probes were removed from the forehead, and an active or sham laser (at 800 or 850 nm) was delivered on either lateral side of the forehead of a participant. Comparisons of (C) correlation coefficients and (D) slopes between the pre-tPBM (blue) and post-tPBM (orange) measurements from 26 young adults in endogenic (right column), neurogenic (middle column), and myogenic (right column) frequency. Error bars are based on the SEM from each respective group. *: p < 0.05; **: p <0.01 by Student’s t-test.

As the second application of quantifying HbT-CSF coupling in this study, we utilized the same 2bbNIRS setup (Figs. 2A and 2B) and algorithms to determine time series of the six parameters (presented in Figs. 2C and 2D) of the PFC of 26 young adults in response to unilateral laser tPBM on the either side of the forehead. The tPBM protocol and stimulation sites on the forehead are illustrated in Figs. 4A and 4B, respectively. The 2bbNIRS data acquisition lasted 7 minutes during the pre- and post-tPBM period, while the 2bbNIRS device was removed during 8-min tPBM for the needed space on the forehead for light delivery.

Following the same data processing steps and statistical analysis as those used for the first application (i.e., studying the AD brain), we obtained group-level linear correlation coefficients (Fig. 4C) of HbT-CSF coupling in the E, N, and M bands during the 7-min pre- (blue) and post-tPBM (orange) phases. tPBM was executed using an 800-nm or 850-nm laser applied to either the right (R) or left (L) forehead (Fig. 4B). Specifically, after analyzed the metrics derived from two lateral tPBM, we had three sub-sets of statistical comparisons in each of the E/N/M bands: 2 sets obtained from 800-nm tPBM delivered on the right and left forehead (800-R and 800-L) and 1 set from 850-nm on the right forehead (850-R). A paired t-test between the pre- and post-tPBM measurements revealed that in 7 out of 9 comparisons (Fig. 4C), tPBM at either wavelength (800 or 850 nm) and on either side of the forehead did not significantly alter the negative correlation coefficients (i.e., coupling strength) between HbT-CSF coupling of the PFC of the young adults.

However, group-level linear correlation slopes of HbT-CSF coupling were significantly enhanced in all three E, N, and M bands by the 8-min tPBM (Fig. 4D). Specifically, in the E band (left panel of Fig. 4D), tPBM in all three cases (800-R, 800-L, and 850-R) enabled a significantly steeper HbT-CSF slope with respect to the pre-tPBM slopes. Moreover, both 800-R and 800-L tPBM also facilitated a significantly steeper hydrodynamic slope in the N band (middle panel of Fig. 4D), while the 850-nm laser significantly strengthened the slope steepness in the M band (right panel of Fig. 4D). In short, these results revealed that tPBM on the forehead boosted a steeper negative correlation in HbT-CSF coupling, despite its effect being somewhat wavelength- and frequency-specific.(15) A stronger negative HbT-CSF slope implies an increased direct impact of HbT alterations on the CSF circulation dynamics. Our findings support the results of animal studies (17–19), demonstrating that tPBM can enhance and stimulate glymphatic clearance dynamics with promising potential for treating patients with AD.

### Comparisons of HbT-CSF coupling among young and older adults

**Figure 5:**
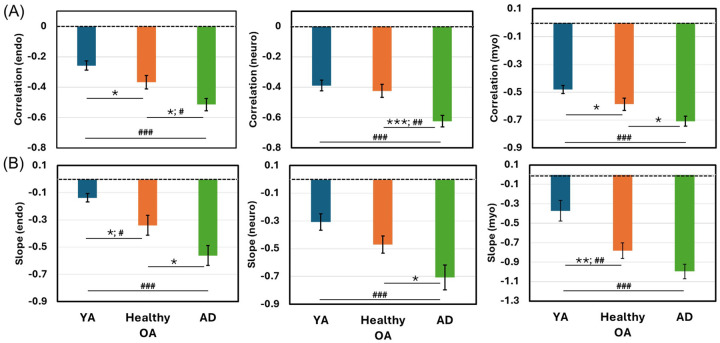
Comparisons of correlation coefficients and slopes across different groups (A) Correlation coefficients and (B) correlation slopes of HbT-CSF coupling in the PFC of healthy young adults (n=26; blue), healthy older adults (n=27; orange), and AD patients (n=16) in three frequency bands: the endogenic (E; 0.005–0.02 Hz; left column), neurogenic (N; 0.02–0.04 Hz; middle column), and myogenic (M; 0.04–0.1 Hz; right column) bands. Error bars represent SEM for each group. Statistical significance was determined using two-sample Student’s t-tests, using marks as follows. *: *p* < 0.05, **: *p* < 0.01, and ***p < 0.001. Additionally, to control for multiple comparisons, we applied the Bonferroni correction, adjusting the significance thresholds to *p* < 0.0167 (0.05/3), *p* < 0.003 (0.01/3), and *p* < 0.0003 (0.001/3), as marked by #, ##, and ###, respectively.

The linear correlation R values and slopes of HbT-CSF coupling obtained during the pre-tPBM period from young adults (n=26) were equivalent to those obtained from the resting PFC of the respective participant group. Thus, we pooled and averaged the respective correlation R and slope values over the three separate pre-tPBM measurements of young adults and plotted the derived metrics in Figures 5(A) and 5(B) along with those from older adults with and without AD for clearer comparisons (i.e., Figs. 3(C) and 3(D)).

Figure 5 shows unambiguously increasing trends of both the correlation R and slope values from young adults to healthy older adults and then to patients with AD across all three infraslow oscillation E/N/M frequency bands. Specifically, age-related increases in both R and slope are significant in only endogenic and myogenic oscillations, which are directly associated with cerebrovascular-CSF dynamics, as marked by ‘*’ and ‘**’. Even after applying Bonferroni correction to control for multiple comparisons among all three groups, the differences in the metrics between young adults and patients with AD were still highly significant.

### tPBM-altered correlation between Δ[oxiCCO] and CSF dynamics

**Figure 6:**
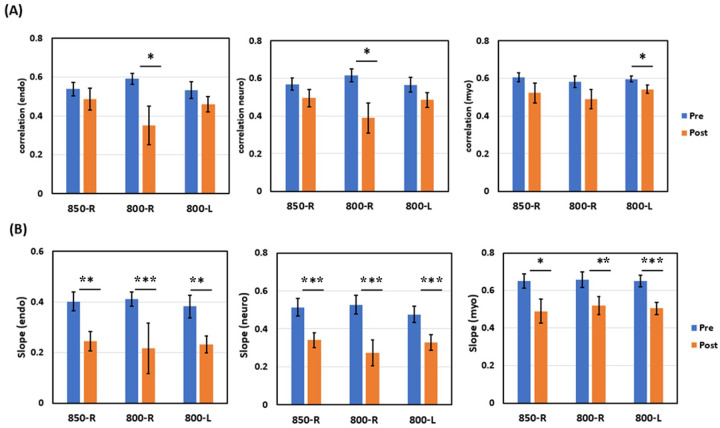
tPBM-altered coupling between metabolic and CSF dynamics in the healthy brain Correlation coefficients and (B) slopes of linear fitting between D[oxiCCO] and D[H_2_O_free_] obtained from the pre-tPBM (blue) and post-tPBM (orange) measurements from 26 young adults in endogenic (right column), neurogenic (middle column), and myogenic (right column) frequencies. Error bars are based on the SEM from each respective group. *: p < 0.05; **: p < 0.01; and ***: p < 0.001 by Student’s t-test.

Because bbNIRS permits specific quantification of Δ[oxiCCO] as compared to regular NIRS meausrements (13, 23, 24), we also calculated the correlation coefficients and slopes of linear fitting between Δ[oxiCCO] vs. Δ[H_2_O_free_] (Fig. 6) in the human PFC in response to tPBM. Specifically, Fig. 6 illustrates that all three tPBM cases on the forehead facilitated a significant reduction in the correlation slope between Δ[oxiCCO] and Δ[H_2_O_free_] in all the E/N/M bands. While changes in the correlation coefficient were not too consistent over different stimulation conditions, 800-nm (but not 850-nm) laser stimulations seemed to create significant decreases in correlation R values.

## DISCUSSION

This study demonstrates the feasibility of quantifying cerebrovascular-CSF coupling in the PFC of human participants using advanced analysis of bbNIRS measurements. Two key biomedical applications are highlighted as examples: (1) characterizing cerebrovascular-glymphatic dynamics in patients with AD compared to age-matched controls and young adults, and (2) examining how tPBM alters correlations among metabolic (Δ[oxiCCO]), cerebrovascular (Δ[HbT]), and glymphatic (CSF) dynamics. The experimental findings for these two specific applications are discussed in detail below.

### Characterization of Cerebrovascular-CSF coupling in older adults with and without AD

The Monro-Kellie hypothesis states that the total volume of the brain, CSF, and cerebral blood (CBV) remains constant (6, 7). Given a fixed intracranial space bounded by a rigid skull, dynamic alterations in the volumes of the CSF and CBV are expected to be inversely correlated with a negative *R*, as reported in ref. (7). In our study, we observed not only negative *R* values but also negative slopes between Δ[HbT] and CSF correlations in older adults with and without AD (Fig. 3). Furthermore, our results demonstrated that patients with AD exhibited tighter correlations and steeper slopes in the cerebrovascular-CSF coupling across all E/N/M bands compared to healthy older adults.

These findings imply that the cerebrovascular-CSF coupling mechanisms are altered in the AD brain. These changes can be attributed to several factors. One of them is associated with AD-related structural changes in the human brain, including overall brain atrophy, increased CSF volume, and reduced CBV (25, 26). Second, the loss of intracranial compliance in patients with AD means that even small changes in CBV might cause larger changes in CSF oscillations, strengthening the relationship. Finally, the overall conditions of AD, such as vascular stiffness, amyloid-related disruption of neurovascular function, and glymphatic impairment, could amplify the effects of changes in CBV on CSF oscillations. All of these may result in higher apparent coupling and steeper slope, even if the relationship is pathological rather than efficient. Moreover, disruptions in CBV-CSF coupling may be indicative of impaired glymphatic function, a hallmark of AD pathology in which the clearance of metabolic waste products is reduced or diminished (27).

Our findings align with prior research, suggesting that AD progression is accompanied by impaired cerebrovascular function and reduced glymphatic clearance efficiency (28). In addition, we expect that a larger negative correlation and/or steeper slope of CBV-CSF coupling might occur only at the early stage of AD, as the brain tries to compensate for emerging dysfunctions. When the brain approaches the late stage of AD, a weaker correlation and shallower slope are expected because of the widespread breakdown of the regulatory mechanisms. Thus, a tighter *R* value and steeper negative slope could indicate early compensatory responses or local dysfunction in the AD brain. This might provide insight into disease staging and help distinguish between regions of compensatory activity and irreversible damage.

Furthermore, Fig. 5 illustrates an age-dependent increase in negative correlations (both R and slope) between cerebral Δ[HbT] and CSF. This observation can be explained by the following physiological mechanisms. As the human brain ages, it undergoes atrophy, thus reducing cerebral blood volume. Consequently, CSF volume increases because the intracranial space is bounded by a rigid skull. The combination of a decrease in Δ[HbT] and an increase in CSF resulted in a more pronounced inverse relationship between them. Thus, our results provide evidence that both the correlation R values and slopes between Δ[HbT] and CSF may serve as new or supplementary features or markers to characterize brain health and the early stages of AD.

### Effects of tPBM on CBV-CSF Coupling

Recent animal studies have reported that tPBM using a 1267-nm laser or 1065-nm LED can activate MLVs for glymphatic clearance of beta-amyloid from the mouse brain (17, 18) or enhance the drainage system of the animal brain during sleep (19). In the second application of this study, we investigated whether tPBM could improve glymphatic dynamics in the human brain. The results revealed that deliveries of tPBM by an 800-nm laser on the either unilateral forehead or by an 850-nm laser on the right forehead enhanced negative correlation slopes between cerebrovascular-CSF coupling in the PFC of the young adults studied, particularly in the endogenic oscillation band (Fig. 4D), while the correlation coefficients of the cerebrovascular-CSF coupling were significantly altered to be more negative in only two out of nine cases (Fig. 4C).

In principle, the correlation R and correlation slope between two variables reflect the relationship between them, but the R and slope values capture different and independent aspects of the relationship. The R value quantifies the strength of the linear relationship and indicates how closely the two variables follow a linear pattern, not their scale. On the other hand, the slope value describes the rate of change, namely, how much the Y variable changes for a one-unit increase in the X variable. The sign of the slope matches that of R, but their magnitudes and meanings are different. Accordingly, a significant increase in the negative correlation slope induced by tPBM, as shown in Fig. 4D, implies that after any of the three tPBM stimulations, the CSF oscillations would be enhanced with a larger amplitude in response to a given oscillation in the CBV or HbT, strengthening the CSF flow and glymphatic dynamics in an antiphase manner. Beyond significant improvements in mitochondrial and cerebral hemodynamic activities (13–15, 29), this study revealed that tPBM may concurrently and efficiently boost the driving mechanism of CSF outflow in the perivascular-CSF interstitial space, thereby promoting glymphatic circulation and enhancing waste clearance from the brain.

**Figure 7:**
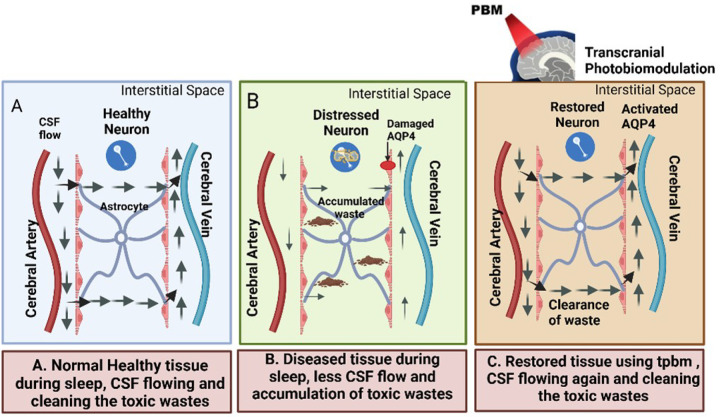
Illustration of the glymphatic system and the effects of tPBM Center panel: A schematic of major anatomical context of the human brain. (A) Schematic of a glymphatic system in healthy neuronal tissue, where CSF flows (dark arrows) through the perivascular-CSF interstitial space efficiently, particularly during sleep (30, 31), facilitating the clearance of neurotoxic wastes. (B) Schematic of a glymphatic system in distressed neuronal tissue, commonly observed in pathological conditions such as Alzheimer’s disease, where impaired CSF flow leads to the accumulation of toxic wastes. (C) Schematic of the restoration of glymphatic function following tPBM, which enhances CSF flow, activates Aquaporin-4 channels (AQP4), and improves the clearance of accumulated wastes.

These observations align with prior research proposing tPBM as a potential intervention for improving cerebral hemodynamics and glymphatic function in patients with AD (30–32). Specifically, several recent studies have presumed differences in the glymphatic system between healthy and diseased tissues (AD brain) of the human brain, particular during sleep (30, 31). Following the rationale or explanations provided in Ref. (30), we present a schematic (Fig. 7) that associates CSF flow with waste clearance efficiency in the brain. Panels A and B of Fig. 7 provide graphical comparisons of the glymphatic systems between normal and AD brains during sleep.

Panel C of Fig. 7 illustrates how tPBM is expected to restore impaired glymphatic function of the human brain by enhancing CSF flow and activating aquaporin-4 (AQP4) channels in astrocytes.^(30)^ This proposed process promotes the clearance of accumulated waste products and neurotoxins, thereby addressing the key pathological features of AD. The improved CBV-CSF coupling post tPBM observed in this study offers strong evidence for this mechanism, demonstrating that tPBM delivered during the awake state can still improve CSF circulation and enhance brain waste clearance (33). Whether tPBM administered during sleep can promote better human glymphatic dynamics (30–32) remains to be confirmed by human studies in the near future.

Figure 5 clearly illustrates that the correlation slope between HbT and CSF or HbT-CSF coupling increases as the human brain ages. Figure 4 shows that tPBM enhances this coupling toward the state of an aging brain. To avoid such discrepancies, it is worth pointing out that tPBM-stimulated promotion of HbT-CSF coupling is dynamic with a short or acute effect, which temporally triggers human glymphatic dynamics and accelerates CSF circulation. In contrast, the data shown in Fig. 5 characterize the human glymphatic state in the resting brain as a neurophysiological metric that reflects the general brain health. Thus, a short-term dynamic intervention, such as tPBM, to boost CSF circulation is a positive and beneficial strategy for improving brain health.

### Advances using bbNIRS to investigate glymphatic dynamics

Previously reported methods used to demonstrate an inverse linear correlation between normalized Δ[HbT] and Δ[H_2_O_free_] were based on regular NIRS using 3–4 NIR wavelengths (6, 7), whereas we employed bbNIRS with 121 wavelengths in this study. Several key points regarding the wavelength selection are worth highlighting. The main reason for using bbNIRS is to accurately quantify Δ[oxiCCO], which has a much lower concentration than other key chromophores (i.e., HbT and water) in living tissues. To determine dynamic patterns of Δ[H_2_O_free_] for CSF, a wavelength near 980 nm (one of the absorption peaks of H_2_O) is necessary to obtain a spectral signature of water in the recorded data (6, 7). If no wavelength representing water features is available in the optical measurement, bbNIRS is an excellent choice for accurately measuring Δ[H_2_O_free_] in living tissues because abundant wavelengths (> 100 wavelengths) can improve the accuracy of water content quantification, even if the water absorption is much lower than that of oxy- and deoxy-hemoglobin within the given spectral region.

Furthermore, with bbNIRS, we had the opportunity to investigate a relationship between cerebral metabolic (Δ[oxiCCO]) and glymphatic dynamics (Δ[H_2_O_free_]) altered by tPBM. Figure 6 shows positive correlation coefficients and slopes between Δ[oxiCCO] and Δ[H_2_O_free_] during the pre- and post-tPBM periods. Specifically, we observed highly significant reductions in positive correlation slopes between the two intervention conditions, suggesting several underlying physiological mechanisms.

First, tPBM is known to directly enhance mitochondrial activity and boosts ATP production. During the pre-tPBM period, the increase in the redox state of CCO (Δ[oxiCCO]) depended on oxygen delivery. During the post-tPBM period, Δ[oxiCCO] became less reliant on fluctuations in oxygen supply, which is highly associated with CSF-related perfusion changes, causing a reduced correlation with CSF dynamics. Second, tPBM has also been shown to enhance microcirculation and vasodilation through nitric oxide release. This enhancement likely stabilizes the oxygen supply and reduces the natural variability in CBF and CSF pulsations. With more consistent oxygen delivery, Δ[oxiCCO] becomes less sensitive to CSF-driven perfusion dynamics, weakening their correlation. Third, with tPBM-enhanced mitochondrial function, the brain may become more efficient at oxygen utilization. This could lead to higher Δ[oxiCCO] levels, even with lower oxygen delivery, making Δ[oxiCCO] less dependent on CSF-driven vascular changes. Finally, tPBM’s direct impact on mitochondrial metabolism may decouple the usual tight relationship between oxygen supply and demand (34). Even if CSF dynamics reflect changes in blood flow and pressure, Δ[oxiCCO] is more reflective of enhanced metabolic efficiency than supply driven oxygenation, which could weaken the observed correlation.

### Selections of wavelengths for tPBM and for quantification of HbT-CSF coupling

It is important to point out the optimal selection of wavelengths for tPBM methodology and NIRS devices to achieve effective research results and the best treatment outcomes. While tPBM using 800–1064 nm has been reported to stimulate Δ[oxiCCO](13–15) (29) and Δ[H_2_O_free_] (in this study), wavelengths near 980 nm, 1065 nm (19), or longer than 1200 nm (17, 18) would be a more efficient choice. These wavelengths are at or near water absorption peaks, and thus they can stimulate the dynamics of MLVs efficiently and lead to the acceleration of CSF circulation. Along the same optical principle, a regular NIRS device using 3–4 NIR wavelengths (6, 7) can be efficient and accurate for determining the correlation metrics between normalized Δ[HbT] and Δ[H_2_O_free_] if at least one of the NIR wavelengths is within the range 900–980 nm, which contains a characteristic absorption feature of water. If one desires to quantify all hemodynamic, metabolic, and glymphatic dynamics, a broadband (e.g., 100 wavelengths) NIRS device covering a wavelength range from 750 to 1000 nm is the most appropriate, with or without tPBM intervention.

### Implications from the two applications combined

Figure 7 emphasizes the critical role of the glymphatic system in maintaining brain health, which facilitates the clearance of neurotoxic waste products and is disrupted in neurodegenerative conditions, such as in the AD brain. On the other hand, tPBM has shown promise in modulating glymphatic or CSF dynamics by enhancing cerebrovascular and glymphatic coupling and functions. The ability of tPBM to improve CBV-CSF coupling and restore glymphatic flow presents a compelling opportunity for developing non-invasive therapies for AD and other neurodegenerative disorders. Future research should focus on investigating the long-term effects of tPBM, particularly its ability to enhance glymphatic circulations and improve cognitive functions.

### Limitations of the study and future work

Although the findings of this study are encouraging, there are several limitations. Technically, we did not utilize a short-separation probe for each bbNIRS channel, so we could not remove contaminants from the superficial layers (i.e. the scalp and skull). All correlation metrics of cerebrovascular-CSF coupling in our study could be underestimated because of the unremoved effects from the extracranial tissues. For the first application (i.e. investigation of the AD brain), the sample size from patients with AD was relatively small, thus reducing the statistical power and generalizability of the results. In addition, we did not perform any cognitive assessments, such as the Mini-Mental State Examination (MMSE), to screen and evaluate the severity of AD in the patient group. While we subjectively estimated that all our AD participants were at an early stage, we did not have any objective measures to confirm this estimation. For the second application (i.e., examination of tPBM effects), the removal of 2bbNIRS probes during tPBM sessions may introduce a potential source of variability in the post-stimulation data, which may impact the interpretation of the results. Moreover, tPBM using an 850-nm laser was conducted only on the right forehead, not on the left lateral side. Thus, it was difficult to thoroughly compare the tPBM-induced effects of the 800- and 850-nm lasers.

For future research, it would be technically advantageous and desirable to implement a short- and long-separation probe in bbNIRS measurement settings to remove signal contamination from extracranial layers. To confirm the characteristics of CBV-CSF coupling in glymphatic dynamics in the AD brain, studies need to recruit and measure a larger and more diverse group of participants, including individuals at various stages of cognitive decline. Also, standard cognitive assessments (e.g., MMSE) should be obtained from each participant for objective associations between the measured CBV-CSF coupling metrics and cognitive measures. Regarding the 2nd application, future research should focus on investigating the long-term effects of tPBM along with standard cognitive assessments to demonstrate its ability to sustain glymphatic function and improve cognitive outcome. The continued effort to refine and expand tPBM as an advancing therapeutic strategy holds significant promise for the treatment of neurodegenerative diseases, including AD.

## MATERIALS AND METHODS

### Study participants and details

For the first application, namely, to study the AD brain, we recruited 43 older adults from the Dallas–Fort Worth community, with 30 healthy participants and 16 patients with AD (8 males, 8 Females: mean age ± SD = 72.8 ± 8.3 years). The inclusion and exclusion criteria for both healthy older adults and patients with AD are shown in Supplementary Material A. Data from 16 patients with AD confirmed by a doctor’s report were included in the analysis. For the second application, namely, to investigate tPBM effects on glymphatic dynamics, 31 healthy young adults were recruited from the university community. For both studies, the Institutional Review Board of the University of Texas at Arlington approved all experimental procedures. All measurements were conducted with informed consent from each participant.

### Experiment setup and protocol for healthy OA and patients with AD

For the first application, the 2bbNIRS experimental setup and protocol followed those detailed in ref. (23). The system was employed to capture NIR spectral alterations in the bilateral forehead of each participant. Two bbNIRS channels, each with a 3-cm source-detector separation, were positioned on the lateral sides of the forehead after each participant sat comfortably (Figs. 2A and 2B). Resting-state data were collected using 2bbNIRS for 14 min, consisting of two phases: an initial 7-minute eyes-open condition followed by a 7-minute eyes-closed condition. In this study, data from only the eyes-closed condition were analyzed.

### Experiment setup and protocol for healthy young adults

For the second application, the setup of 2bbNIRS was the same as that used in the first application (Figs. 2A and 2B) during the 7-min pre- and post-tPBM measurements. Each experiment involved three phases: a 7-min pre-stimulation phase, an 8-min randomized tPBM/sham stimulation phase, and a 7-min post-stimulation phase, lasting 22 minutes in total (13, 15, 23), as illustrated in Fig. 4A. During the stimulation phase, the two bbNIRS probes were removed, while the laser stimulation was delivered to either lateral forehead (Fig. 4B). Each participant attended five sessions or visits spaced at least seven days apart to avoid residual tPBM carry-over effects. The five visits consisted of the following tPBM conditions: 800-nm tPBM on the (1) right (800-R) or (2) left (800-L) forehead, (3) 850-nm tPBM on the right (850-R) forehead, and sham stimulation on the (4) right and (5) left forehead.(15) Sequences of the five experiments were randomly assigned to each subject. During each experiment, the participants sat comfortably and kept their eyes closed without falling asleep. Laser protection googles were worn for eye protection (Fig. 4B).

### Data Analysis

Since the bbNIRS system was highly sensitive to motion artifacts, several subjects with excessive motion during the experiments were excluded from the analysis. After exclusion, the raw bbNIRS data from 26 healthy young adults (14 males, 12 females; mean age ± SD = 22.4 ± 2.3 years) were used in this study. For healthy older adults, data from 27 out of 30 was used (5 males, 22 females; mean age ± SD = 67 ± 5.6 years).

For the 2nd application (tPBM effects), the raw bbNIRS data taken from multiple visits were previously reported in our recent publication (15) that focused on bilateral hemodynamic or metabolic connectivity and unilateral hemodynamic-metabolic coupling altered by tPBM in the young human brain. In this study, we reanalyzed the same raw data from three visits with only true active tPBM, with the emphasis on tPBM-induced glymphatic dynamics and its linear coupling with cerebrovascular alterations (i.e., HbT).

**Figure 8:**
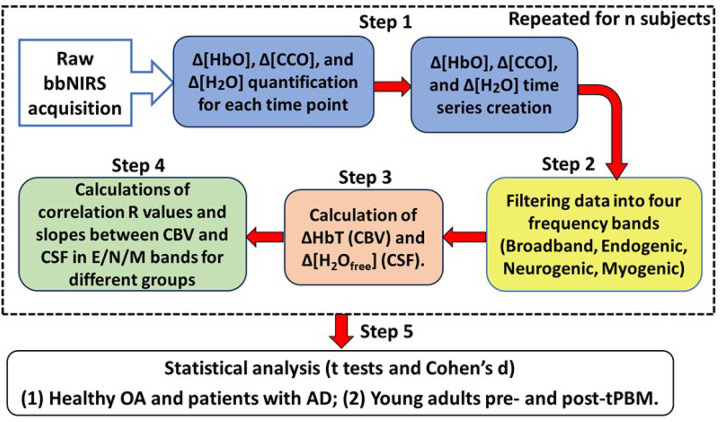
A data processing flowchart with five steps. Step 1: Quantify Δ[HbO] or Δ[HbT], Δ[oxiCCO], and Δ[H_2_O] at each time point, followed by construction of time series for each respective metric or variable (blue boxes). Step 2: Filter each time series, Δ[HbT], Δ[oxiCCO], and Δ[H_2_O], into four infraslow frequency ranges in the broadband, endogenic (E), neurogenic (N), and myogenic (M) bands (yellow box). Step 3: Calculate the frequency-specific time series of Δ[HbT] (representing CBV) and Δ[H_2_O_free_] (representing CSF); the latter is obtained by subtracting the normalized Δ[HbT] from the normalized Δ[H_2_O] (orange box). Step 4: Determine the linear correlation coefficients and slopes between Δ[HbT] and Δ[H_2_O_free_] in each E/N/M band (green box) with the averaged data between both lateral sides of the forehead. The dashed large box outlines the four steps repeated for the respective participants and the respective time periods in the two applications. For example, in Application 2, Steps 1 to 4 were repeated for 7-min pre- and post-tPBM periods under each of the three tPBM conditions (800-R, 800-L, and 850-R). Step 5: Statistical testing for significant differences in each neurophysiological parameter in each E/N/M band between (1) healthy OA vs. patients with AD and (2) pre- and post-tPBM measurements in young adults.

The algorithm used to quantify Δ[HbO], Δ[Hb], and Δ[oxiCCO] of living tissue or the human cortex has been published in several studies (35, 36) including ours (13, 23, 24). The newly developed or advanced algorithm used in this study is the addition of calculations of the water content in the PFC, Δ[H_2_O], and the corresponding free water content in the CSF, Δ[H_2_O_free_]. Figure 8 shows a flowchart outlining the overall data-processing procedure, with steps describing the derivation of the newly added Δ[H_2_O_free_] quantities.

#### STEP 1: *Conversion of ΔOD(t, λ) to Δ[HbO](t), Δ[oxiCCO](t) and Δ[H*_*2*_*O](t) over time*

In bbNIRS data acquisition, the sampling rate was 0.67 Hz (1.5 sec per spectrum), resulting in 280 temporal points in 7-min measurement period. Each data point represents a NIR spectrum (740 to 1100 nm) with a spectral interval of 0.38 nm (13, 15, 23). The first data processing step was to convert the raw spectral data within 780–900 nm, which were included in the 2bbNIRS readings, to Δ[HbO], Δ[Hb], Δ[HbT] (= Δ[HbO] + Δ[Hb]), Δ[oxiCCO], and Δ[H_2_O] at each time point by adding the water component as a contributor to the NIR spectral absorption in the modified Beer-Lambert law. Accordingly, a relative optical density spectrum, ΔOD(t, λ), is defined and calculated at each wavelength λ at each time *t* as:

1 $$\Delta OD(t,\lambda )=log_{10}[\frac{I_{0}(t=0,\lambda )}{I(t,\lambda )}],$$

where *I*_*0*_*(t = 0, λ)* is the baseline spectrum at time *t* = 0 or an average of several initial baseline spectral readings, and *I(t, λ)* represents time-resolved NIR spectra acquired at each time point, *t*, throughout the entire experiment duration. A detailed mathematical derivation from the raw NIR spectra to the quantification of Δ[HbO], Δ[Hb], Δ[oxiCCO], and Δ[H_2_O] can be found in refs. (13, 23, 24) and represented graphically in Figure S2. After repeating the calculations for each time point, we obtained the time series of Δ[HbO](t), Δ[Hb](t), Δ[oxiCCO](t), and Δ[H_2_O](t) from ΔOD(t, λ).

#### STEP 2: *Time series of Δ[HbO], Δ[HbT], Δ[oxiCCO], and Δ[H*_*2*_*O] in E/N/M bands*

Subsequently, the time series for Δ[HbO], Δ[HbT], Δ[oxiCCO], and Δ[H_2_O] were filtered to isolate three distinct frequency bands in accordance with infraslow oscillation (ISO) standards, referred to as the E/N/M components, as reported (22, 23) and used in previous studies (15, 21, 34). Figure S3 graphically illustrates the process of decomposition of a Δ[HbO] time series into the three frequency bands, as an example.

#### STEP 3: *Dynamics of free water content in CSF*

In theory, the cerebral concentration of [HbT] is proportional to the total cerebral blood volume or CBV; thus, the oscillation of Δ[HbT] represents that of the CBV. The measured water signal, Δ[H_2_O], results from the intracellular and extracellular spaces within the measurement volume interrogated by bbNIRS optical probes. However, the intracellular water concentration is not expected to fluctuate significantly during a 7-min measurement period in the resting brain. Alterations in the calculated H_2_O content are believed to arise from (1) fluctuations in CSF volume, referred to as cerebral free water, Δ[H_2_O_free_] (6, 7), and (2) fluctuations in the volume of water bound to the blood, which oscillates along Δ[HbT]. Accordingly, quantifications of dynamic oscillations of Δ[H_2_O_free_] in the CSF were achieved by subtracting the normalized Δ[HbT] from the normalized Δ[H_2_O], using the methodology published in Refs. 6 and 7. This calculation strategy was used in both applications of this study.

#### STEP 4: *Linear fitting of correlation coefficients and slopes between CBV and CSF*

To investigate cerebrovascular-CSF coupling in the human PFC in both applications, the time series of Δ[HbT] (as CBV) and Δ[H_2_O_free_] (as CSF) from each lateral measurement of the forehead were linearly fitted to obtain the correlation coefficient, R, and slope in each frequency E/N/M band per participant. The correlation coefficient, R, and slope values from both lateral probes were then averaged to offer a single set of R and slope in each of E/N/M bands for each patient/participant in each of the pre- and post-tPBM periods. Both the R values and slopes between Δ[HbT] and Δ[H_2_O_free_] coupling were expected to be negative because of the Monro-Kellie hypothesis, which states that the total volume of the brain, CSF, and intracranial blood remains constant (6, 7). Both the fitted correlation coefficients and slopes in each of the three frequency bands were obtained at the group level and compared against their appropriate counter groups.

In addition, because bbNIRS has the unique ability to quantify Δ[oxiCCO] compared to regular NIRS(13, 23, 24), we also computed the frequency-specific correlation coefficients and slopes between Δ[oxiCCO] and Δ[H_2_O_free_] in the human PFC for the 2nd application.

#### STEP 5: *Statistical Analysis*

For the 1st application, a two-sample unequal-variance t-test was performed between older adults with and without AD for each correlation coefficient and slope in all E/N/M bands. For the 2nd application, a paired unequal-variance t-test was used to examine significant differences in the correlation metrics between pre- and post-tPBM in young adults. The significance level was set at three thresholds of p < 0.05, 0.01, and 0.001. We also included calculations in Cohen’s *d* to assess the effect size of the statistical significance for the 1st application. Accordingly, 0.2 < *d* < 0.5, 0.5 < *d* < 0.8, 0.8 < *d* < 1.3, and *d* > 1.3 were considered small, medium, large, and very large effect sizes, respectively. The same statistical analysis and thresholds were used to identify significant differences in the correlation metrics between Δ[oxiCCO] vs. Δ[H_2_O_free_] for the 2nd application. Furthermore, Bonferroni correction was used to adjust the significance thresholds when we compared the correlation coefficient R and slope of Δ[HbT] vs. Δ[H_2_O_free_] among young adults, healthy older adults, and patients with AD, with the corrected *p* values of *p* < 0.0167 (0.05/3), *p* < 0.003 (0.01/3), and *p* < 0.0003 (0.001/3).

## Figures and Tables

**Table 1A:** Statistical results of bilaterally averaged correlation coefficients between healthy OA (n = 27) and AD patients (n = 16)

frequency	Endogenic (0.005–0.02 Hz)	Neurogenic (0.02–0.04 Hz)	Myogenic (0.04–0.1 Hz)
**p value**	<0.015	<0.001	<0.032
**Cohen’s d**	−0.76	−1.06	−0.65

**Table 1B:** Statistical results of bilaterally averaged correlation slopes between healthy OA (n = 27) and AD patients (n = 16)

frequency	Endogenic (0.005–0.02 Hz)	Neurogenic (0.02–0.04 Hz)	Myogenic (0.04–0.1 Hz)
**p value**	0.034	0.032	0.052
**Cohen’s d**	−0.66	−0.72	−0.60

## Data Availability

All bbNIRS raw data will be available publicly as of the date of publication. The DOI will be listed in the key resources table. Any additional information required to reanalyze the data reported in this paper is available from the lead contact upon request.
